# Rapid Determination of Six Low Molecular Carbonyl Compounds in Tobacco Smoke by the APCI-MS/MS Coupled to Data Mining

**DOI:** 10.1155/2017/8260860

**Published:** 2017-04-20

**Authors:** Wuduo Zhao, Qidong Zhang, Binbin Lu, Shihao Sun, Shusheng Zhang, Jianxun Zhang

**Affiliations:** ^1^Zhengzhou Tobacco Research Institute, China National Tobacco Corporation, Zhengzhou 450001, China; ^2^College of Chemistry and Molecular Engineering, Zhengzhou University, Zhengzhou 450001, China

## Abstract

A simple method was established for the rapid determination of low molecular carbonyl compounds by the combination of atmospheric pressure chemical ionization tandem mass spectrometry (APCI-MS/MS) and data mining. The ionization was carried out in positive mode, and six low molecular carbonyl compounds of acrolein, acetone, propionaldehyde, crotonaldehyde, butanone, and butyraldehyde were analyzed by both full scan mode and daughter scan mode. To overcome the quantitative difficulties from isomer of acetone/propionaldehyde and butanone/butyraldehyde, the quantitation procedure was performed with the characteristic ion of [CH_3_O]^+^ under CID energy of 5 and 15 eV. Subsequently, the established method was successfully applied to analysis of six low molecular carbonyl compounds in tobacco smoke with analytical period less than four minutes. The contents of acrolein, acetone, propionaldehyde, crotonaldehyde, butanone, and butyraldehyde for a cigarette were about 63 ± 5.8, 325 ± 82, 55 ± 9.7, 11 ± 1.4, 67 ± 5.9, and 12 ± 1.8 *μ*g/cig, respectively. The experimental results indicated that the established method had the potential application in rapid determination of low molecular carbonyl compounds.

## 1. Introduction

Low molecular carbonyl compounds, existing in the cigarette smoke and automobile exhaust, aroused extensive attention due to their adverse effects on human health [1, 2]. Due to the relatively low boiling points and high vapor pressures, low molecular carbonyl compounds were easy to inhale by the human body and cause irritation of the eyes and throat, headache, sickness, and even cancers [3, 4]. Carbonyl compounds, such as acetaldehyde, were listed as air toxics in the Clean Air Act Amendments of 1990. Moreover, the Texas Commission on Environmental Quality (TCEQ) developed inhalation toxicity factors for the evaluation of acrolein and crotonaldehyde concentrations in 2014. In conventional method, the analysis of carbonyl compounds was based on derivatization. Carbonyl compounds reacted to derivatization reagents firstly and then analysis by capillary electrophoresis, chromatography, or chromatography coupled with mass spectrometer [5–9]. However, these analytical methods usually involved derivatization and multistep sample preparation procedure, which were labor-intensive, time-consuming, and tedious.

Mass spectrometry is one of the extensively used techniques for the identification of molecules in complex mixture owing to its universality and accuracy [10–12]. Due to less fragment ion, several online and real-time analyses were established based on the combination of soft ionization source and mass spectrometer, which had been proven as a powerful analytical technique and applied in food safety, air pollution, and biology [13–16]. Atmospheric pressure chemical ionization (APCI) source is one of the important soft ionization sources [17, 18]. The ionization of sample for APCI was carried out through ion-molecule reaction in gas phase, which was more suitable for the direct detection of gas sample. Recently, APCI-MS had been applied in direct monitoring of volatile organic compounds in ambient air [19–21].

In previous study, the commercial APCI source of Xevo™ TQ MS (Waters) was modified to introduce sample directly into the ionization region, and acrolein and crotonaldehyde were investigated by the APCI-MS/MS. However, the isomers in low molecular carbonyl compounds such as acetone/propionaldehyde and butanone/butyraldehyde were difficult to analyze by the APCI-MS/MS due to the same molecular mass. To overcome the difficulty from the isomer, the MS/MS spectra under a series of CID energies were investigated to provide more information, which was in favor of differentiation through data mining. In this study, a method was developed for the rapid determination of low molecular carbonyl compounds by APCI-MS/MS. The quantitation procedure of six low molecular carbonyl compounds was performed with the characteristic ion of [CH_3_O]^+^ under CID energy of 5 and 15 eV. Finally, six low molecular carbonyl compounds in tobacco smoke were detected with APCI-MS/MS to test and prove the developed method.

## 2. Experimental

### 2.1. Chemicals and Reagents

Acrolein, propionaldehyde, crotonaldehyde, and butyraldehyde (99.5%) were purchased from Quality Control Chemicals INC (USA); acetone and butanone (>99%) were purchased from Fisher (UK); deionized/distilled water was made by Thermo Scientific GenPure. Nitrogen gas was purchased from Yuanzheng Technology Company (China). Standards solution was prepared by diluting the 5000 *μ*g/mL stock solutions by water to a final concentration. 10 *μ*L solution was injected into the container to make gas sample with 0.01~5 *μ*g/L. Tobacco smoke (3R4F) was extracted from the tube behind the Cambridge filter about 30 cm on smoking machine provided by Zhengzhou Tobacco Research Institute (China).

### 2.2. Apparatus

To achieve the quantitative analysis of low molecular carbonyl compounds, the standards gas was made by the gasification of standards solution in a 2 L glass container as described in our previous study [21]. In order to clean the container automatically, two solenoid valves were used to control the gas switching, and the flow rate of cleaning gas was 180 L/h.

**Figure 1:**
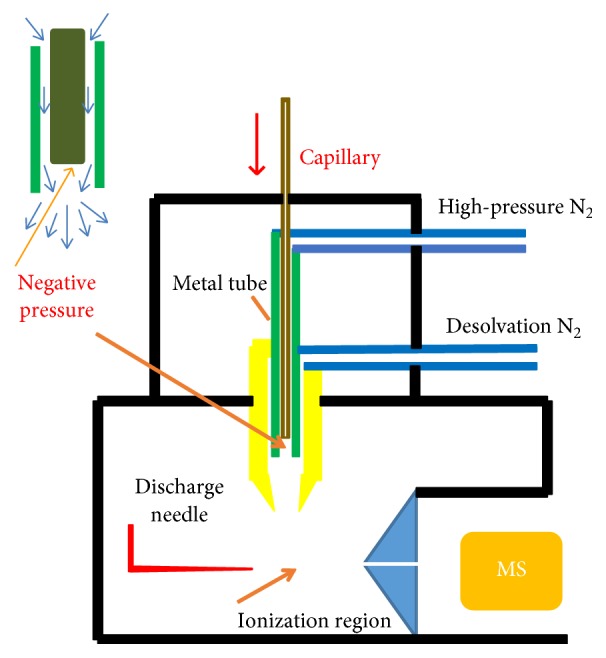
Schematic diagram of modified APCI source.

In order to introduce gas sample directly into the ionization region, APCI source of Xevo™ TQ MS (Waters, USA) was modified and the schematic diagram was shown in Figure 1. The deactivated metal capillary (0.021′′ inner diameter, 0.029′′ outer diameter, Restek, USA) was sheathed by a metal tube with 1 mm inner diameter. High-pressure nitrogen with 0.6 MPa was passed between capillary and metal tube. As nitrogen passed the end of the capillary, the cross section of gas flow was increased abruptly, and a negative pressure was produced due to the Venturi effect. Thus, the sample was introduced into the mass spectrometer ionization region through capillary. The gas flow rate in capillary was about 110 mL/min. To decline the memory effects, the capillary was cleaned by the gas in the container before the sampling. Meanwhile, the sampling volume was about 220 ml (2 min, 110 ml/min), which was larger than that of dead volume in the capillary.

The operating conditions of mass spectrometry were as follows. Ionization mode was positive; discharge current was 4 *μ*A; APCI probe temperature was 100°C; desolvation gas flow was 150 L/h; cone gas flow was 10 L/h; collision gas flow rate was 0.25 mL/min.

## 3. Results and Discussion

### 3.1. Ionization of Six Low Molecular Carbonyl Compounds

**Figure 2:**
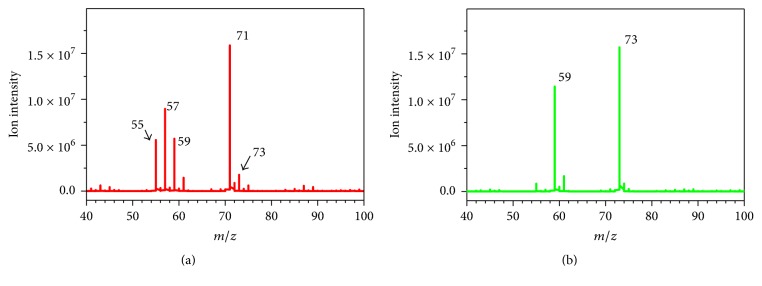
(a) Full scan mass spectra of four aldehyde mixtures with 0.5 *μ*g/L for acrolein, propionaldehyde, crotonaldehyde, and butyraldehyde, respectively. (b) Full scan mass spectra of two ketone mixtures with 0.5 *μ*g/L for acetone and butanone, respectively.

The full scan mass spectra for the mixture of four aldehydes and mixture of two ketones with 0.5 *μ*g/L were displayed in Figure 2, respectively. Peaks at* m/z* 57, 59, 71, and 73 were observed in Figure 2(a), which were corresponding to the [MH]^+^ of acrolein, propionaldehyde, crotonaldehyde, and butyraldehyde, respectively. Peaks at* m/z* 59 and 73 were observed in Figure 2(b), which were corresponding to the [MH]^+^ of acetone and butanone, respectively. The generation of [MH]^+^ was ascribed to proton transfer reaction, where the proton affinities (PA) of low molecular carbonyl compounds were higher than that of water. In Figure 2(a), peak at* m/z* 55 was ascribed to C_4_H_7_^+^, which was produced by the loss of H_2_O from the protonated butyraldehyde.

**Figure 3:**
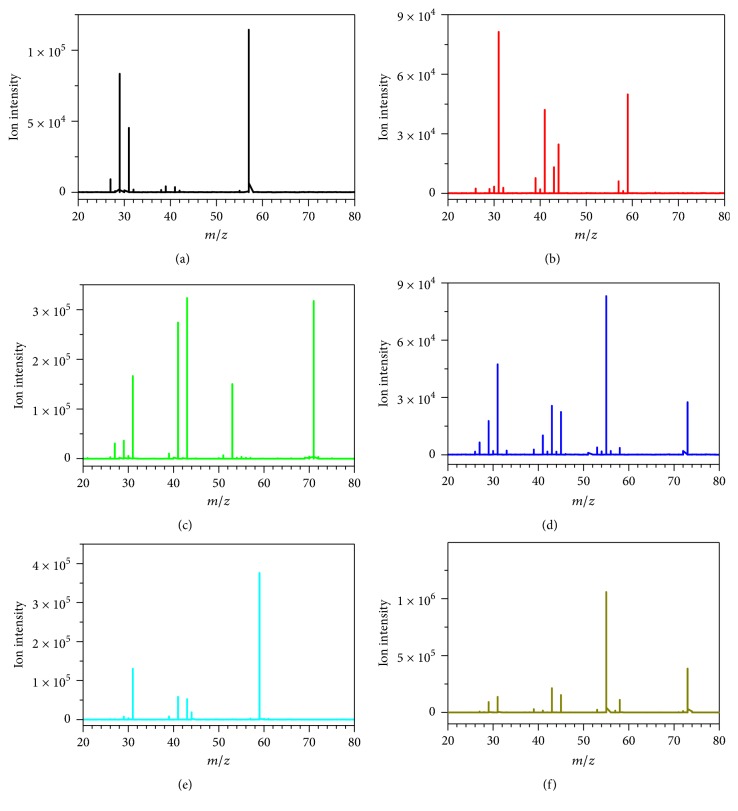
Daughter scan mass spectra of six low molecular carbonyl compounds with 0.5 *μ*g/L under the CID energy of 15 eV. (a) Acrolein, (b) propionaldehyde, (c) crotonaldehyde, (d) butyraldehyde, (e) acetone, and (f) butanone.

Daughter scan mode was usually used to find characteristic fragment ions for quantitative analysis. The daughter scan mass spectra of low molecular carbonyl compounds under CID energy of 15 eV were shown in Figure 3. As for low molecular carbonyl compounds, the carbonyl group was activated by the ionization through proton transfer reaction, and H_2_O (*m/z* 57→39,* m/z* 59→41,* m/z* 71→53, and* m/z* 73→55) or CO (*m/z* 57→29,* m/z* 71→43, and* m/z* 73→45) was easy to lose in the collision cell [22, 23]. As for acetone/propionaldehyde, although the protonated aldehydes were easy to lose CO, the formation of* m/z* 31 was not likely produced through the loss of CO due to the instability of C_2_H_7_^+^ (*m/z* 31) in Figures 3(b) and 3(e). Therefore, the ion of* m/z* 31 was ascribed to [CH_3_O]^+^ which was formed by the C-C band break. In Figure 3,* m/z* 31 was observed in all the daughter scan mass spectra of six low molecular carbonyl compounds, which was ascribed to [CH_3_O]^+^. Due to the relation of [CH_3_O]^+^ with carbonyl group, [CH_3_O]^+^ was used as characteristic ion for quantification.

### 3.2. Quantification of Isomer Carbonyl Compounds

**Figure 4:**
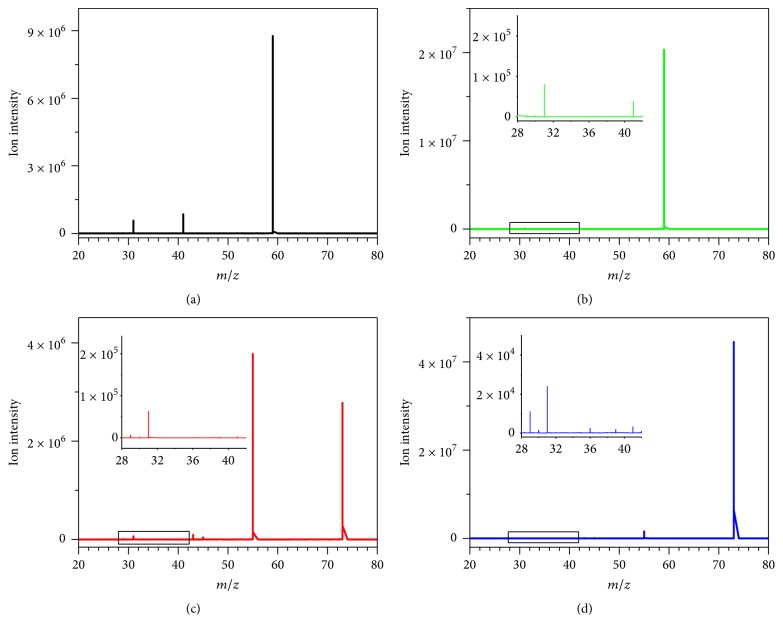
Daughter scan mass spectra of 0.5 *μ*g/L isomers of low molecular carbonyl compounds under the CID of 5 eV. (a) Propionaldehyde, (b) acetone, (c) butyraldehyde, and (d) butanone.

Due to the same molecular mass, it was difficult to quantify the isomer carbonyl compounds in the mixture without the chromatographic separation. To overcome the quantitative difficulties, more structure information should be obtained to distinguish acetone/propionaldehyde and butanone/butyraldehyde. Due to the different functional groups or positions in the molecule, the analysis of carbonyl isomers could be performed by chromatography coupled with derivatization [24, 25]. However, derivatization methods usually involved multistep sample preparation and long analytical period. MS/MS was usually used to obtain structure information under the selected CID energy, while the plentiful structure information reflected by the CID energy was not in full use. Therefore, the influence of CID energy on the MS/MS spectra was investigated under a series of CID energies, such as 5, 15, 25, and 35 eV. When the CID energy was at 5 eV, there were obvious differences of the fragment intensities (*m/z* 31) in Figure 4 for acetone, propionaldehyde, butanone, and butyraldehyde, which was in favor of differentiation through data mining. As the CID energy was increased to 25 eV, the actual intensities were decreased. Meanwhile, the fragment ion distribution and actual intensity of acetone and butanone were similar to those of propionaldehyde and butyraldehyde. Due to the marked differences in MS/MS spectra for isomers of aldehydes and ketones under 5 and 15 eV, detailed statistical evaluation of data is not needed for discrimination of propionaldehyde/acetone and butyraldehyde/butanone. Therefore, the CID energies of 5 and 15 eV were chosen for quantification of the six low molecule carbonyl compounds.

In Figure 4(a), peaks at* m/z* 31 of propionaldehyde were observed, while this of acetone in Figure 4(b) was markedly lower compared with that of propionaldehyde. When the CID energy was increased to 15 eV, the actual intensity of* m/z* 31 for acetone was increased about 1.6 times in Figures 3(e) and 4(b), while that of propionaldehyde was decreased about 86% in Figures 3(b) and 4(a). When the CID energy was increased from 5 to 15 eV, the actual intensity of* m/z* 31 for butanone in Figures 3(f) and 4(d) was increased about 5.8 times, while that of butyraldehyde was decreased about 25% in Figures 3(d) and 4(c).

The relationship between the ion intensity and the concentration was expressed as *I* = *σ* × *C*. Here, *I* was the ion intensity, *C* was the ion concentration, and *σ* was the response coefficient. The relation between ion intensity at* m/z* 31 and the isomer concentration under 5 and 15 eV could be expressed as follows:

(1) $$\begin{array}{c} \left[\begin{array}{c} I\left(5\;ev\right)\\ I\left(15\;ev\right) \end{array}\right]=\left[\begin{array}{cc} \sigma_{1}^{}\left(5\;ev\right) & \sigma_{2}^{}\left(5\;ev\right)\\ \sigma_{1}^{}\left(15\;ev\right) & \sigma_{2}^{}\left(15\;ev\right) \end{array}\right]\times \left[\begin{array}{c} C_{1}\\ C_{2} \end{array}\right]. \end{array}$$

Here, *σ*_1_(*n*) and *σ*_2_(*n*) were the response coefficient of* m/z* 31 under the CID energy of *n* for two isomers, respectively; *C*_1_ and *C*_2_ were the concentration of the two isomers in the mixture. *I*(*n*) was the measured ion intensity at* m/z* 31 for the mixture under the CID energy of *n* in the mass spectra.

### 3.3. Linear Calibration Curves

Table 1 showed the calibration curves function of six low molecular carbonyl compounds by daughter scan mode with fragment ion [CH_3_O]^+^ under the CID energy of 5 and 15 eV. With the use of the standard definition of S/N = 3, the detection limit of butyraldehyde was up to 0.003 *μ*g/L. Meanwhile, Figure S1 of supplementary information available online at https://doi.org/10.1155/2017/8260860 showed the calibration curves with the concentration of 0.01, 0.025, 0.05, 0.1, 0.5, 1.0, 2.5, and 5 *μ*g/L. It showed a good linear relationship between the ion intensity and concentration, and the response coefficients were extracted from the slope of the calibration curve.

Acetone and propionaldehyde mixture (1 : 1) with 0.5 *μ*g/L in the container was prepared using the weighting method to test the reliability of quantification based on (1). The errors between the concentration calculated by (1) and the actual concentration calculated by weighting method ranged from −12% to 5% for acetone and from −8% to 3% for propionaldehyde. Moreover, butanone and butyraldehyde mixture (1 : 1) with 0.5 *μ*g/L was also measured. The errors between the concentration calculated by (1) and the actual concentration calculated by weighting method ranged from −3% to 1% for butanone and from −10% to 2% for butyraldehyde, which demonstrated good accuracy achieved by (1).

### 3.4. Measurement of Six Low Molecular Carbonyl Compounds in Tobacco Smoke

**Figure 5:**
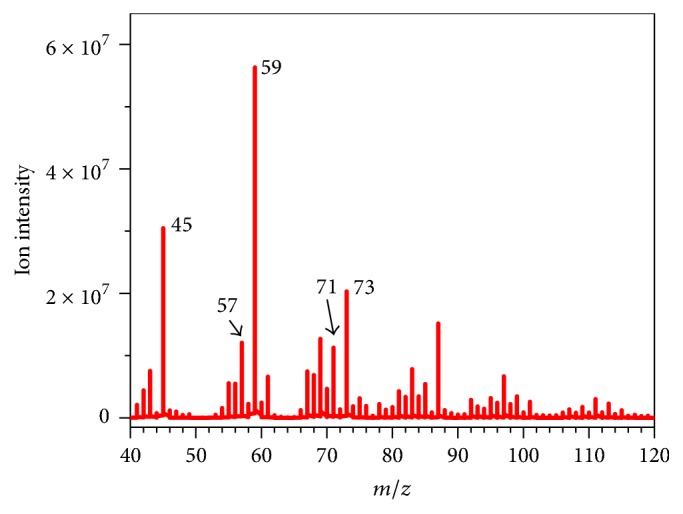
Full scan mass spectrum of tobacco smoke.

Low molecular carbonyl compounds have been found as the combustion product of cigarette. In this study, 5 mL main stream smoke of cigarette extracted from the smoke machine was injected into the container, and 10 *μ*L water was injected into the container at the same time. After two minutes, the tobacco smoke was analyzed by the APCI-MS/MS. The full scan mass spectrum of tobacco smoke was shown in Figure 5; low molecular carbonyl compounds of acetaldehyde (*m/z* 45), acrolein (*m/z* 57), crotonaldehyde (*m/z* 71), acetone/propionaldehyde (*m/z* 59), and butanone/butyraldehyde (*m/z* 73) were observed clearly.

The daughter scan mass spectra of low molecular carbonyl compounds were shown in Figure S2 of supplementary information. According to the standards curve in Table 1, the value was calculated about 0.57 and 0.1 *μ*g/L for acrolein and crotonaldehyde in container. Based on the intensity change of [CH_3_O]^+^ under the CID energies of 5 and 15 eV, the values for acetone, propionaldehyde, butanone, and butyraldehyde in container were about 2.9, 0.49, 0.6, and 0.11 *μ*g/L through the calculation with (1). The contents of acrolein, acetone, propionaldehyde, crotonaldehyde, butanone, and butyraldehyde for a cigarette were about 63 ± 5.8, 325 ± 82, 55 ± 9.7, 11 ± 1.4, 67 ± 5.9, and 12 ± 1.8 *μ*g/cig (35 mL puff volume, 8 times), which agreed with the result of Wagner et al. and Sampson et al. [26, 27]. The contents of acrolein and crotonaldehyde for a cigarette were different between this paper and [21] (57 and 9.5 *μ*g/cig); the error of the analysis was ascribed to two aspects. One is the instrument stability, which was about ±5%, and the other is the reproducibility of cigarette smoke produced by the combustion, which was about ±10%. The different result was ascribed to the statistical difference. The determination of six low molecular carbonyl compounds in tobacco smoke was less than four minutes, and the agreement of results further verified that the developed method was reliable for the determination of the low molecular carbonyl compounds.

## 4. Conclusion

Six low molecular carbonyl compounds of acrolein, acetone, propionaldehyde, crotonaldehyde, butanone, and butyraldehyde in the cigarette smoke were analyzed rapidly through the combination of APCI-MS/MS and data mining. As for the isomers of carbonyl compounds, the quantification was performed by the APCI-MS/MS under two CID energies of 5 and 15 eV. A quantitation procedure was developed for low molecular carbonyl compounds with characteristic ion of [CH_3_O]^+^. The capability of the developed method was demonstrated through quantification of six low molecular carbonyl compounds in tobacco smoke with analytical period less than four minutes.

## Supplementary Material

Linear calibration curves for six low molecular carbonyl compounds.

## Figures and Tables

**Table 1:** Performance parameters of the APCI-MS/MS method for six low molecular carbonyl compounds by daughter scan mode with [CH_3_O]^+^.

Analytes	Linear equation	Linear range (*μ*g/L)	LOD	*R* ^2^	CID energy
Acrolein	*y* = 7.45*e*4*x* + 1.76*e*2	0.025~2.5	0.007	0.9979	15 eV
Acetone	*y* = 1.95*e*5*x* + 2.3*e*3	0.05~5	0.021	0.9973	15 eV
Propionaldehyde	*y* = 1.37*e*5*x* + 9.93*e*2	0.025~2.5	0.008	0.9993	15 eV
Crotonaldehyde	*y* = 3.53*e*5*x* + 5.62*e*2	0.01~2.5	0.004	0.9998	15 eV
Butanone	*y* = 2.13*e*5*x* + 1.3*e*3	0.05~5	0.012	0.9997	15 eV
Butyraldehyde	*y* = 6.47*e*4*x* + 7.54*e*2	0.025~2.5	0.006	0.9976	15 eV
Acetone	*y* = 1.25*e*5*x* + 9.88*e*2	0.05~5	0.009	0.9996	5 eV
Propionaldehyde	*y* = 1.02*e*6*x* + 1.58*e*3	0.01~2.5	0.003	0.9993	5 eV
Butanone	*y* = 3.26*e*4*x* + 4.71*e*2	0.05~5	0.013	0.9986	5 eV
Butyraldehyde	*y* = 1.21*e*5*x* + 4.06*e*1	0.025~2.5	0.003	0.9995	5 eV
