# Supplementary material for: Rapid Determination of Six Low Molecular Carbonyl Compounds in Tobacco Smoke by the APCI-MS/MS Coupled to Data Mining
Source: J Anal Methods Chem. 2017 Apr 20;2017:8260860. doi: 10.1155/2017/8260860 (PMC5415865; doi:10.1155/2017/8260860)
Supplement: Supplementary file 1 — Linear calibration curves for six low molecular carbonyl compounds. [file 8260860.f1.doc]

### Rapid determination of six low molecular carbonyl compounds in tobacco smoke by the tandem mass spectrometry coupled to atmospheric pressure chemical ionization

Wuduo Zhao ^a,b^, Qidong Zhang ^a^, Binbin Lu ^a^, Shihao Sun ^a^, Shusheng Zhang ^b,*^ and Jianxun Zhang ^a^,*

*^a^Zhengzhou Tobacco Research Institute, China National Tobacco Corporation, Zhengzhou 450001, China*

*^b^College of Chemistry and Molecular Engineering, Zhengzhou University, Zhengzhou 450001, China*

**E-mail:* [zsszz@126.com](mailto:zsszz@126.com); [jxzh258@126.com](mailto:jxzh258@126.com).


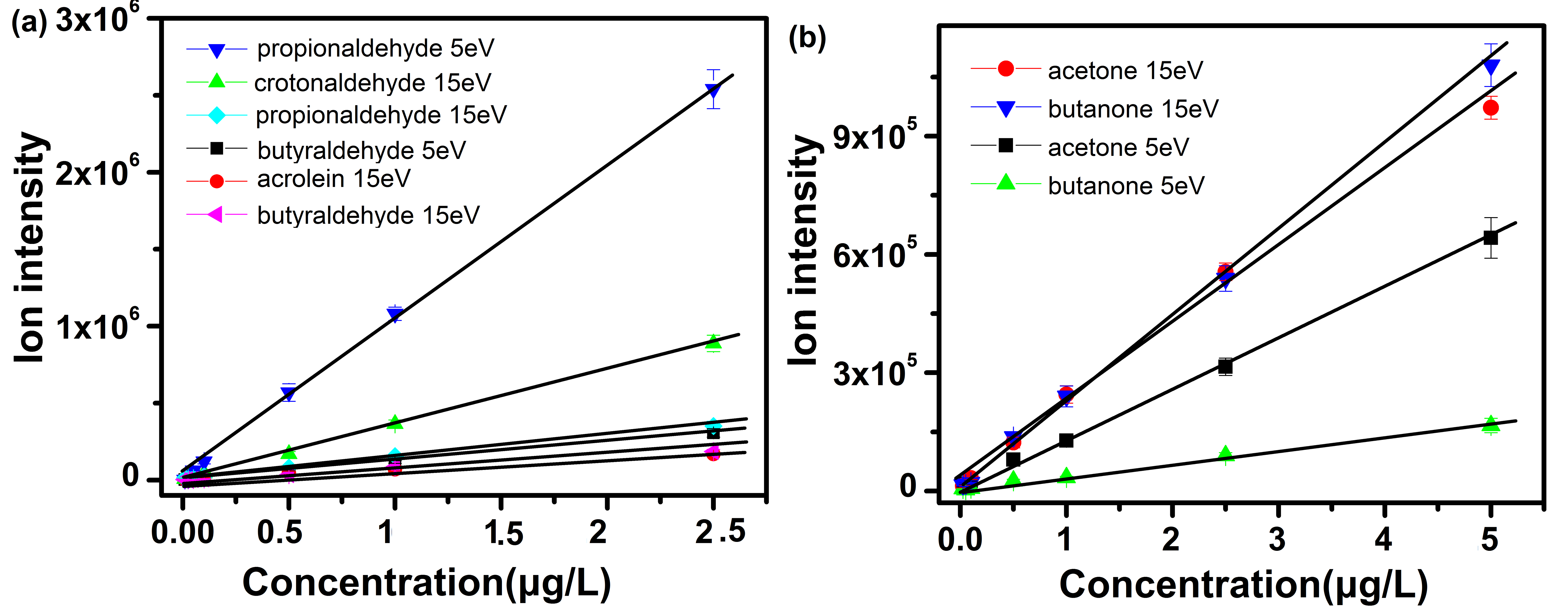


**FIGURE S1:** (a) Linear calibration curves for acrolein 15 eV, propionaldehyde 15 V, crotonaldehyde 15 eV, butyraldehyde 15 eV, propionaldehyde 5 eV, and butyraldehyde 5 eV with the concentration range from 0.01 to 2.5 μg/L. (b) Linear calibration curves for acetone 15 eV, butanone 15 eV, acetone 5 eV and butanone 5 eV with the concentration range from 0.025 to 5 μg/L.


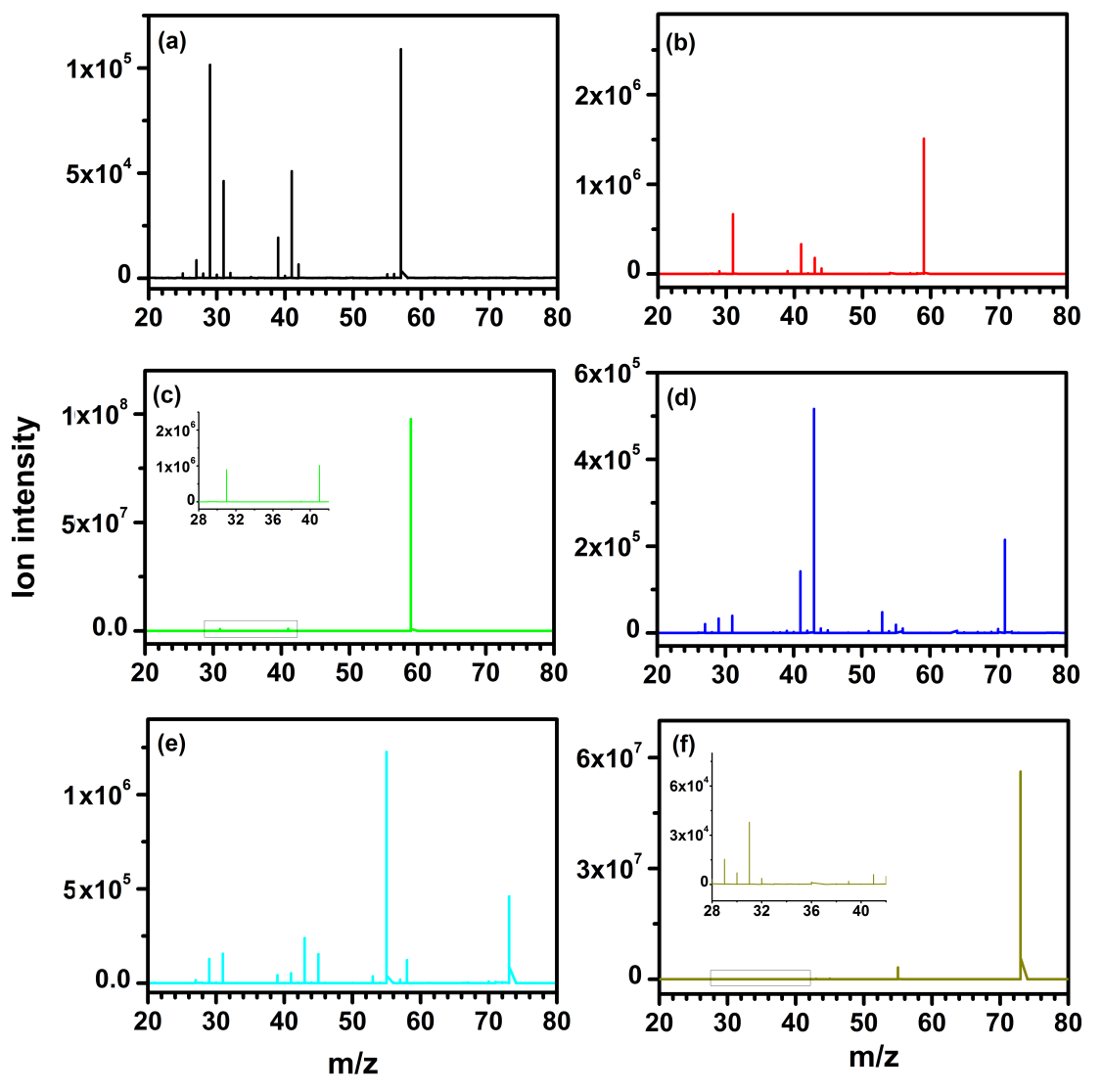


**FIGURE S2:** Daughter scan mass spectra of six low molecular carbonyl compounds in tobacco smoke. (a) acrolein 15 eV (b) acetone/propionaldehyde 15 eV (c) acetone/propionaldehyde 5 eV (d) crotonaldehyde 15 eV (e) butanone /butyraldehyde 15 eV and (f) butanone/butyraldehyde 5 eV.
